# Fragile Histidine Triad (FHIT) Suppresses Proliferation and Promotes Apoptosis in Cholangiocarcinoma Cells by Blocking PI3K-Akt Pathway

**DOI:** 10.1155/2014/179698

**Published:** 2014-03-16

**Authors:** Qiang Huang, Zhen Liu, Fang Xie, Chenhai Liu, Feng Shao, Cheng-lin Zhu, Sanyuan Hu

**Affiliations:** ^1^Department of General Surgery, Qilu Hospital of Shandong University, No. 107 Wenhuaxi Road, Jinan 250012, China; ^2^Department of General Surgery, Anhui Provincial Hospital Affiliated with Anhui Medical University, Hefei, Anhui 230001, China; ^3^Hepatic-Biliary-Pancreatic Key Laboratory of Anhui Province, Hefei, Anhui 230001, China

## Abstract

Fragile histidine triad (FHIT) is a tumor suppressor protein that regulates cancer cell proliferation and apoptosis. However, its exact mechanism of action is poorly understood. Phosphatidylinositol 3-OH kinase (PI3K)-Akt-survivin is an important signaling pathway that was regulated by FHIT in lung cancer cells. To determine whether FHIT can regulate this pathway in cholangiocarcinoma QBC939 cells, we constructed an FHIT expression plasmid and used it to transfect QBC939 cells. Protein and mRNA expression were measured by western blotting and qRT-PCR, respectively. The viability and apoptosis of QBC939 cells were then assessed using MTT assays and flow cytometry. Our results revealed that the expression of survivin and Bcl-2 was downregulated, and caspase 3 was upregulated, in cells overexpressing FHIT. In addition, FHIT suppressed the phosphorylation of Akt. The changes in cell proliferation and apoptosis were obvious in cells overexpressing FHIT which parallels that of treatment with LY294002, a potent inhibitor of phosphoinositide 3-kinases. Treatment with LY294002 further decreased the expression of survivin and Bcl-2 and increased caspase-3 levels. These results suggest that FHIT can block the PI3K-Akt-survivin pathway by suppressing the phosphorylation of Akt and the expression of survivin and Bcl-2 and upregulating caspase 3.

## 1. Introduction

Cholangiocarcinoma is a highly aggressive malignancy, with an extremely poor prognosis. Currently [1], surgical resection is the only curative treatment for this devastating disease. However, most cancers of the biliary tract have grown beyond the limits of curative resection by the time they become clinically evident. Although most cholangiocarcinoma patients have a poor prognosis after surgical resection, some patients have a more favorable postoperative course. Therefore, an improved understanding of the molecular mechanisms associated with the progression of cholangiocarcinoma is important and may be beneficial for the development of novel effective therapeutic strategies.

A growing body of evidence suggests that specific fragile sites in human chromosomes play roles in tumorigenesis. Research has focused on FHIT. FHIT is altered in many different kinds of primary or advanced carcinomas. Specifically, deletions within both FHIT alleles result in the loss of exons with a concomitant absence of full-length FHIT transcript and protein. Loss of expression of the FHIT gene with a loss of regulatory control is common in epithelial malignancies. The FHIT gene expression has been found in primary tumors and cell lines derived from lung, breast, head and neck, esophagus, stomach, colon and rectum, pancreas, kidney, cervix, and liver cancer [2–5]. Our previous studies showed that the loss of FHIT protein may play an important role in the carcinogenesis and prognosis of cholangiocarcinoma [6].

Although data suggest that FHIT is a tumor suppressor gene, its exact mechanism of action and the signaling pathways it activates are poorly understood. PI3K-Akt pathway was an important pathway which could promote proliferation and inhibit apoptosis [7, 8]. Therefore, we speculated that FHIT-induced apoptosis occurs via the inactivation of the PI3K-Akt signaling pathway in cholangiocarcinoma. The aim of this study is to investigate whether FHIT plays an important role in the development and prognosis of cholangiocarcinoma through the inactivation of the PI3K-Ak signaling pathway.

## 2. Materials and Methods

### 2.1. Cell Culture and Antibodies

The human cholangiocarcinoma QBC939 cell line was obtained from the Cell Bank of the Chinese Academy of Sciences (Shanghai, China). It was cultured in RPMI 1640 (Sigma, St. Louis, MO, USA) medium supplemented with 10% fetal bovine serum and penicillin and streptomycin (Sigma) at 37°C in a humidified 5% CO_2_ atmosphere. Rabbit polyclonal antibodies against FHIT were purchased from Life Technologies (USA). Rabbit polyclonal antibodies against survivin, Bcl-2, caspase-3, AKT and p-AKT were from Genscript Co. (USA).

### 2.2. Construction of FHIT Plasmids and Transfection

Human FHIT cDNA was amplified from total RNA isolated from QBC939 cells (a human cholangiocarcinoma cell line) by RT-PCR using the primers 5′-ccaatggatccATGTCGTTCAGATTTGGCCA-3′ and 5′-ccaatctcgagTCACTGAAAGTAGACCCGCAGA-3. The PCR product was cloned into the pcDNA3.1 plasmid (Life Technologies). The sequences of all constructs were confirmed by sequencing. The transfection of plasmids into QBC939 cells was performed using Lipofectamine (Life Technologies), and positive clones were selected using neomycin. The cells were divided into four groups: a wild-type group (cell), a negative control group (NC; transfected with empty vector), an experimental group that was transfected with the FHIT overexpression plasmid (FHIT), and an LY294002 (PI3K-Akt signal pathway inhibitor) treatment group that was treated with 20 *μ*mol LY294002 for 48 h.

### 2.3. Real-Time PCR (RT-PCR)

QBC939 cells were cultured and transfected with FHIT plasmid or empty vector. RT-PCR was performed to detect the expression of* FHIT*,* survivin*,* Bcl-2*, and* caspase-3*. Total RNA was extracted from tumor cells using TRIzol reagent (Life Technologies). Next, 2 *μ*g total RNA was reverse-transcribed using a First Strand cDNA Synthesis Kit (Promega, USA) to synthesize cDNA. RT-PCR was then used to analyze the expression of* FHIT*,* survivin*,* Bcl-2,* and* caspase-3* using 2 *μ*L cDNA and SYBR Green Master Mix (Bio-Rad, USA) following the manufacturer's instructions. The following primers were used: FHIT forward 5′-GAGTCGGGACAGTGGTGGA-3′ and reverse 5′-GCTTCTGCTGCCATTTCCTC3′; survivin forward 5′-AGTCTGGCGTAAGATGATGGAT-3′ and reverse 5′-GTGCAT-TTTCAGTTGTTTCTGC-3′; Bcl-2 forward 5′-ACTTGTGGCCCAGATAGGCACCCAG-3′ and reverse 5′-GCACTTCGCCGAGATGTCCAGCCACCAG-3′; caspase-3 forward 5′-TCCACCACCCTGTTGCTGTAG-3′ and reverse 5′-GACCACAGTCCATGACATCACT-3′; and GAPDH forward 5′-TCATCACCATTGGCAATGAG-3′ and reverse 5′-GTGTTG GCGTACAGG-3′. GAPDH was used as an internal control. The PCR products were loaded onto 2% agarose gels and visualized using ethidium bromide staining under ultraviolet light. Each PCR product was analyzed in triplicate, and the threshold cycle (Ct) values of the target genes were normalized to the endogenous control. Differential expression was calculated following the 2^−ΔΔCt^ method.

### 2.4. Western Blotting

Cells were harvested and lysed in radioimmunoprecipitation assay (RIPA) buffer (20 mM Tris-HCl pH 7.5, 150 mM NaCl, 10 mM EDTA, 1 mM EGTA, 1% nonidet P-40, 0.5% sodium deoxycholate, and 0.1% sodium dodecyl sulfate). Protein concentrations were calculated using a BCA protein assay kit (Thermo Fisher Scientific, USA), and equal amounts of total protein per lane were separated by SDS gel electrophoresis and transferred to polyvinylidene fluoride membranes (Sigma, USA) using semidry transfer. Nonspecific binding of proteins to the membrane was blocked by incubation with TBS-T buffer (50 mM Tris-HCl pH 7.4, 150 mM NaCl, and 0.1% Tween-20) containing 5% skimmed milk. Membranes were then incubated with the appropriate primary and horseradish peroxidase-conjugated secondary antibodies. The primary rabbit polyclonal antibodies were all purchased from Cell Signaling Technologies (Beverly, MA, USA). Western blot images were captured using an Epi Chemi II Darkroom and Sensicam imager with Labworks 4 software (UVP; PerkinElmer Life Science, Boston, MA, USA).

### 2.5. Cell Proliferation Assay

QBC939 cells were seeded into 96-well plates (3,000 per well) in a final volume of 200 *μ*L. After a 24 h attachment period, cells were treated with LY294002, and media were replaced every 24 h. Cells were treated as indicated, and cell proliferation was assessed using the 3-(4,5-dimethylthiazol-2-yl)-2,5-diphenyltetrazolium assay (MTT, Sigma) daily. The optical density (OD) values were measured at 490 nm using a microplate spectrophotometer (Molecular Devices, Sunnyvale, CA, USA). The proliferation index, calculated as the absorbance of cells from the different treatment group compared with control, was determined.

### 2.6. Annexin V-FITC/PI Staining

Cells in the different groups were treated as indicated, and Annexin V-FITC/PI assays were performed following the manufacturer's protocol (Roche Diagnostics, Pleasanton, CA, USA). Briefly, cultured cells were harvested, washed with binding buffer, and incubated in 200 *μ*L binding buffer containing 5 *μ*L of Annexin-V-FITC, and the nuclei were counterstained with PI (propidium iodide). The percentage of apoptotic cells was determined using a FACSCalibur flow cytometer (Becton-Dickinson Immunocytometry Systems, San Jose, CA, USA).

### 2.7. Statistical Analysis

All statistical analyses were performed using SPSS ver. 13.0 software (SPSS Inc., Chicago, IL, USA). Pearson's chi-squared (*χ* ^2^) test or Fisher's exact test was used to analyze the differences between groups. Data were presented as the mean ± standard deviation, and the differences between groups were assessed using analysis of variance (ANOVA) or Dunnett's *t*-test. A *P* value of <0.05 was considered to indicate statistical significance.

## 3. Results

### 3.1. Overexpression of FHIT

**Figure 1:**
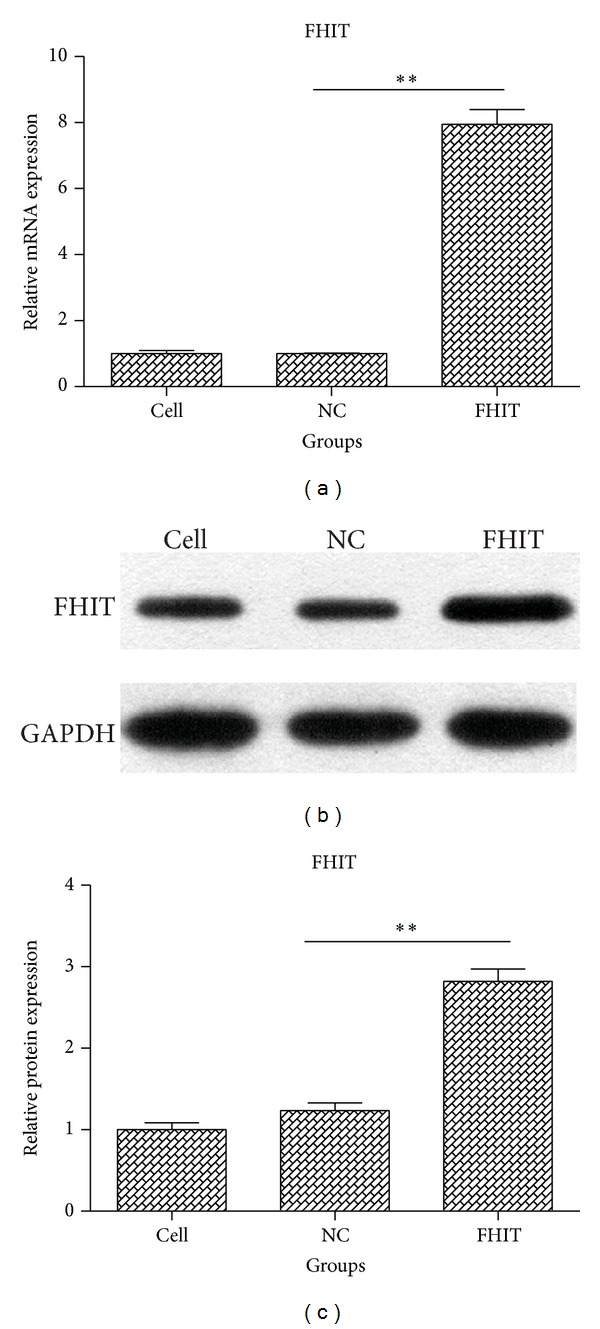
The expression of FHIT in QBC939 cells after transfection. (a) The expression of* FHIT* was assessed in each group after transfection using quantitative real-time RT-PCR. (b) Western blotting for FHIT protein expression. (c) Quantification of FHIT protein expression in each group presented in bar graphs as fold-increase. **P < 0.01.

To investigate the function of FHIT, we constructed an FHIT overexpression vector and transfected it into QBC939 cells. The expression of FHIT was then confirmed using RT-PCR and western blotting. The mRNA expression of FHIT was increased 7.945-fold after transfection (Figure 1(a)), and the protein levels were also increased (Figure 1(b)). As shown in Figure 1(c), the protein levels of FHIT were increased 2.1-fold compared with empty vector-transfected QBC939 cells. These data suggest FHIT was successfully overexpressed in QBC939 cells after transfection with the FHIT overexpression plasmid.

### 3.2. Levels of p-Akt Were Suppressed by FHIT

**Figure 2:**
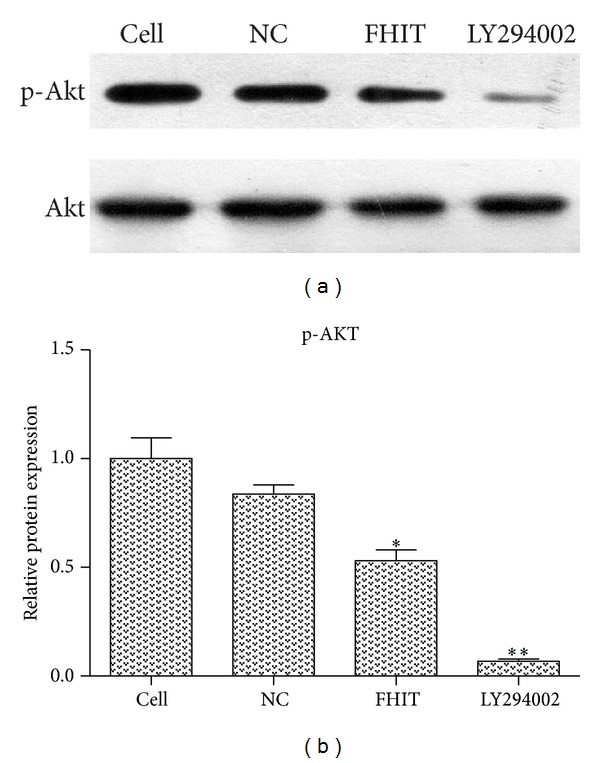
The levels of p-Akt were assessed by western blotting. (a) Western blotting for p-Akt. (b) Quantification of p-Akt protein levels in each group, presented in bar graphs as fold-increase. *P < 0.05 versus NC/FHIT; **P < 0.001 versus NC/LY294002.

To determine the role of FHIT in the PI3K-Akt pathway, we investigated the levels of p-Akt in cells transfected with the FHIT expression vector. Western blotting revealed that the levels of p-Akt were suppressed in QBC939 cells treated with LY294002 (the PI3K inhibitor) or transfected with FHIT (Figure 2). These data suggest that FHIT could suppress the phosphorylation of Akt.

### 3.3. The Expression of Survivin, Bcl-2, and Caspase-3 in FHIT-Overexpressing Cells

**Figure 3:**
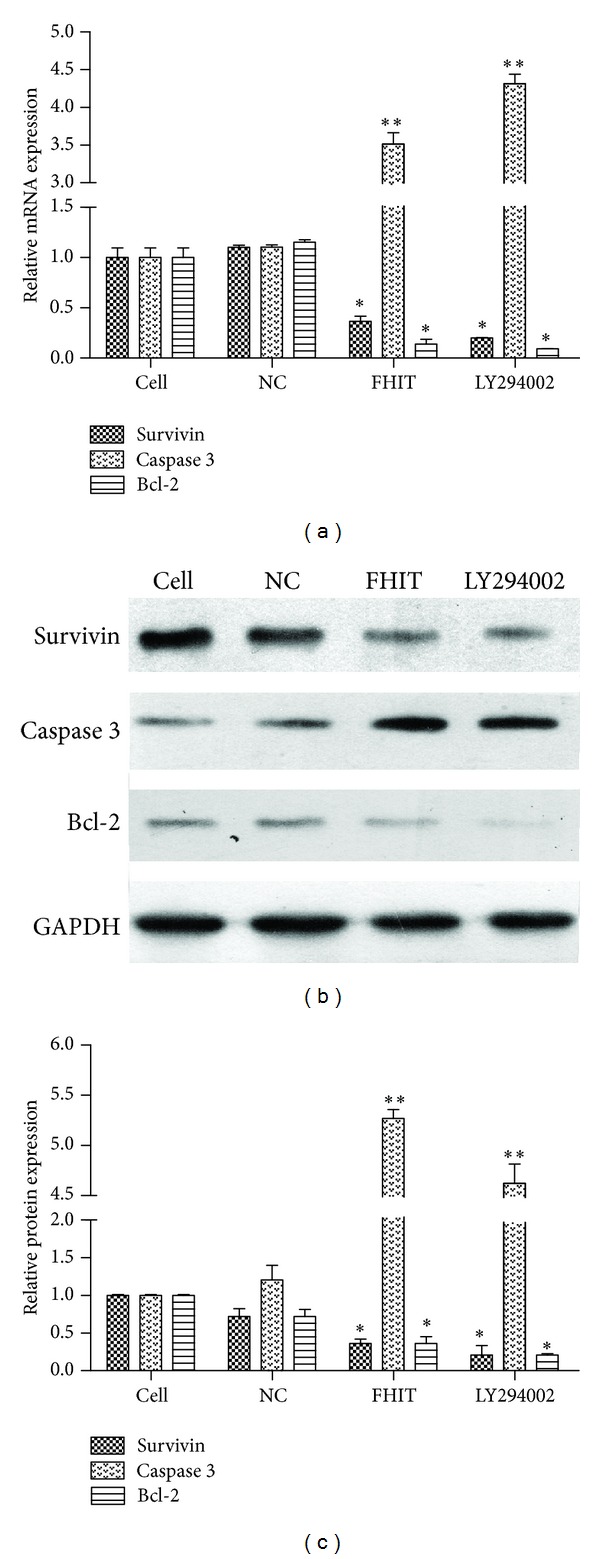
The expression of survivin, Bcl-2, and caspase-3 in QBC939 cells in each group after transfection or treatment with LY294002. (a) The mRNA expression of* survivin*,* Bcl-2*, and* caspase-3* in each group, as assessed by RT-PCR. (b) Western blotting for survivin, Bcl-2, and caspase-3. (c) Quantification of the expression levels of survivin, Bcl-2, and caspase-3 in each group, presented in bar graphs as fold-increase. *P < 0.05, **P < 0.01.

RT-PCR analysis revealed that* survivin* mRNA levels were decreased 2.7-fold,* bcl-2* levels were decreased 7.2-fold, and* caspase-3* expression was increased 3.514-fold in FHIT-overexpressing cells compared with either control group (Figure 3(a)). These data were confirmed by analyzing the protein expression of survivin, Bcl-2, and caspase-3. Data revealed thatthe levels of survivin and Bcl-2 were downregulated, and the levels of caspase-3 were upregulated (Figure 3(b)). As shown in Figure 3(c), the levels of survivin and Bcl-2 were decreased 4.8-fold and 5.9-fold, respectively. And caspase-3 protein expression increased 5.2-fold (Figure 3(c)). The expression of survivin, Bcl-2, and caspase-3 was also assessed in cells treated with LY294002 as a positive control. The expression profiles were similar to those observed after FHIT overexpression, as shown in Figures 3(a)–3(c). The mRNA and protein expression levels of survivin and Bcl-2 mRNA were downregulated, and caspase-3 was upregulated in LY294002-treated cells.

### 3.4. Cell Proliferation Was Suppressed and Apoptosis Was Promoted in Cells after the Overexpression of FHIT

**Figure 4:**
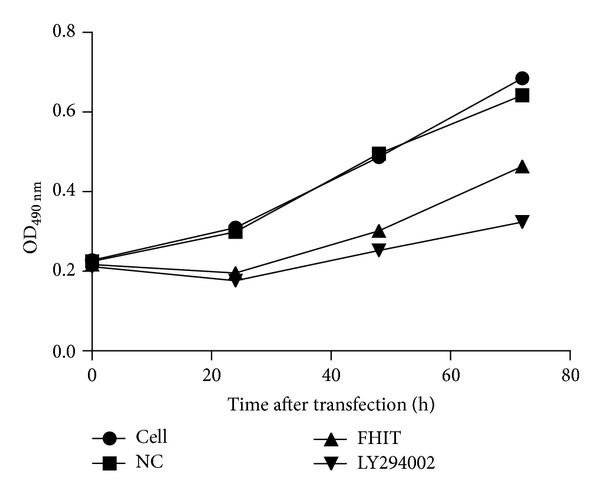
Effect of FHIT overexpression and LY294002 treatment on cell proliferation. OD (optical density) was used to assess cell proliferation at 0, 24, 48, and 72 h after treatment with LY294002 or transfection.

**Figure 5:**
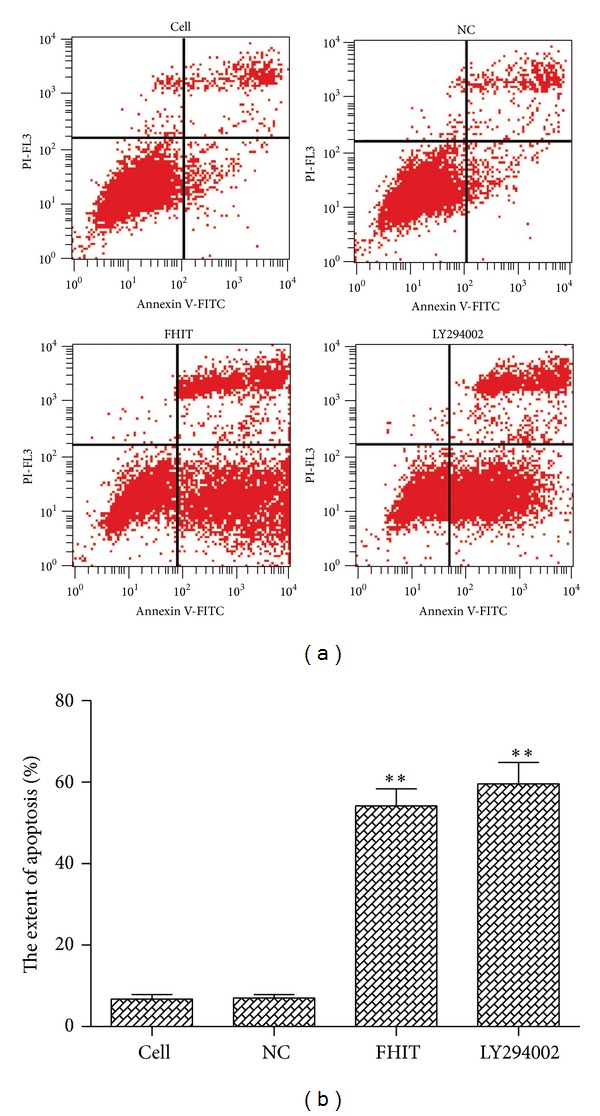
Apoptosis in each group was assessed after transfection or treatment with LY294002. Apoptosis was assessed after 48 h treatment with 20 *μ*mol LY294002. **P < 0.01.

Cell proliferation and apoptosis were determined by MTT assay and flow cytometry, respectively. MTT assay revealed that cell proliferation was reduced in cells overexpressing FHIT or treated with LY294002 compared with the NC group (Figure 4). Consistent with this, cellular apoptosis was increased in the FHIT-overexpressing and LY294002 treatment groups (Figure 5). These data suggest that both FHIT and LY294002 could inhibit growth and promote apoptosis in QBC939 cells.

## 4. Discussion

The FHIT gene spans the most common fragile site in the human genome, FRA3B (3p14.2) [9]. This region frequently undergoes biallelic loss, cytogenetic abnormalities, and genomic rearrangement in tumors [10, 11]. Recently, studies revealed that the adenoviral overexpression of FHIT in human pancreatic cancer cells effectively suppressed cell growth and induced caspase-dependent apoptosis in both* in vitro* and* in vivo* experiments [12]. In addition, FHIT protein deficiency resulted in altered sensitivity to mitomycin C, UVC, and ionizing radiation in human gastric carcinoma cells in clonogenic assays [13]. Our previous study showed that FHIT could suppress cholangiocarcinoma cells growth and promote apoptosis.

PI3K/Akt pathway was an important pathway which could promote proliferation and inhibit apoptosis. Meanwhile, survivin, Bcl-2, and caspase-3 were the downstream target of Akt, which were related with proliferation and apoptosis [14, 15]. These strongly remind us that FHIT may affect downstream gene through the PI3K/Akt pathway and thus plays a role as tumor suppressor. So in this study, we further study the possible mechanism of molecular biology.

We investigate the potential pathway in which FHIT regulates cells proliferation and apoptosis in cholangiocarcinoma. We found that the expression of p-Akt was decreased after FHIT was overexpressed. These observations were comparable to the effects of LY294002, which blocked the PI3K/Akt pathway by combing with inactive PI3K. So we thought FHIT could inhibit the phosphorylation of Akt, but how FHIT block PI3K/Akt pathway was needed to explore.

To confirm the effect of FHIT on PI3K/Akt pathway, we measured the expression of survivin, Bcl-2, and caspase-3 which play a key role in the regulation of cell proliferation and apoptosis. Specially, survivin was one of the members of protein family which could inhibit apoptosis, regulate the cell cycle, and promote angiogenesis [16]. Our study found the expression of survivin in cholangiocarcinoma was higher than normal tissue adjacent to carcinoma and had negative correlation with the expression of FHIT protein by immunohistochemistry (data not shown). Another study suggested that the proapoptotic effect of UA on HepG2 cells is mediated by activation of caspase-3 and is highly correlated with inactivation of PI3K/Akt/survivin pathway [17]. The results of our study showed that the expression of survivin and bcl-2 was decreased and the expression of caspase-3 was increased after FHIT transfection. Importantly, the effects of FHIT overexpression were similar to the LY294002. Then we measured the cell growth and apoptosis by MTT and flow cytometry after the overexpression of FHIT in QBC939 cells. Data revealed that the overexpression of FHIT andthe addition of LY294002 could suppress cholangiocarcinoma cell growth and induce apoptosis in QBC939 cells. All of these results indicated that blocking up PI3K/Akt pathway, as the effect of FHIT, could suppress cholangiocarcinoma cell growth and apoptosis. Moreover, Semba [18] also found FHIT could regulate the expression of survivin via PI3K/Akt pathway in lung cancer. So we declared that the effects of FHIT on proliferation and apoptosis in QBC939 cells were mediated by downregulation of survivin and bcl-2 and the activation of caspase-3 via the PI3K pathway.

In conclusion, the PI3K-Akt pathway may play an important role in FHIT-induced proliferation and apoptosis in cholangiocarcinoma cells. Understanding the relationship between FHIT and PI3K-Akt pathway could provide useful information not only for the early detection of cholangiocarcinoma, but also for the development of novel therapeutic strategies.
